# Synthesis, Optimization, and Characterization of Ecofriendly Production of Gold Nanoparticles Using Lemon Peel Extract

**DOI:** 10.1155/2021/7192868

**Published:** 2021-12-13

**Authors:** Eman Alzahrani, Ashwaq T. Alkhudidy

**Affiliations:** Department of Chemistry, College of Science, Taif University, P.O. Box 11099, Taif 21944, Saudi Arabia

## Abstract

This study examines the importance of utilizing green synthesis using lemon peel for gold nanoparticles over other chemicals since it is environmentally friendly, available, and cheap. Several parameters were optimized to ensure the extraction of the GNPs concentration of lemon peels using HAuCl_4_ and lemon peel extract having a ratio of 2 : 1. For the optimum result, the ratio used was 2 : 1. The gold nanoparticles fabrication happened in 10 minutes. The initial observation was the color change of the solution. The UV-visibility spectroscopic studies are performed to confirm the result. The experiments are done concurrently to ensure the solution is mixed on the proper ratio. The GNP is also characterized by the different techniques in their sizes and electronic transmission microscopy, essential in extracting gold nanoparticles. Other elements of the composition are removed by the EDAX methods, the FTIR method, and the TEM methods, all of which reveal the real reason behind the required extraction capacity. Most gold nanoparticles show a maximum absorption rate at the peak of 535 to 579 nm. The result obtained from the TEM and the SEM analysis revealed that the grain size is analogized to the average size of 6.67 nm. With a simple synthesis of the price, some processes show that the medically available nanoparticles are necessary. The used method in this paper to fabricate GNPs is cheap, easy, fast, and sustainable and it can be done with ease in any laboratory.

## 1. Introduction

“Nano” comes from the Greek word “Nanos,” which depicts a minute subject. This is also used to the billionth prefix to show the magnified size of the particle. According to ASTM International 2006, nanoparticles have two dimensions [1]. The particles have unique chemical and physical characteristics that show the materials' bulkiness to form total products properly. The exposure to the quantum effect is the disruption of the particles, which shows the appropriate shape of the particles [2–11]. Nanoparticles are also different in size since they represent the nature of small materials, which often depict the characteristics of most chemicals [12–16]. Generally, NPs is found in quantum dots, carbon nanotubes, nanocapsules, fullerenes, ceramic NPs, and nanoemulsions [17].

The gold nanoparticles are known as noble metals since they are resistant to rust and oxidation. They also attract attention due to their shiny physical appearance, stability, smallness, and significant biocompatibility [5]. Due to their high surface area to volume ratio, they are easily converted to other forms of affinity to show their commitment to new physical use, especially with their complex matrices, which often play a crucial role [3–5]. The nanoparticles have an attractive appearance with high electrical conductivity and a diverse microorganism. Other characteristics also possess close relations to the nanosized particles, which are turning in shape and can include the morphology of the surface [3].

Different methods to fabricate the gold nanoparticles have been devised to ensure following the rules and preparation methods of the particles [6]. A general system for connecting GNPs joins made physical and average points of view [7]. The standard compound structure for mixing gold nanoparticles wires the usage of shocking facilitated substances, for instance, sodium borohydride [18]. Such strategies are rich, and the hazardous composed materials foster that going with nanoparticles is noncompatible for human use. More lately, nanoparticles have been made using microorganisms [19–21], compounds [22], creatures [23], and plants or plant extracts [24–28] which have been proposed as possible ecofriendly choices as opposed to substance and authentic frameworks. Green science has been applied considering the exact outcomes that the world is trying to achieve along with the limited open opportunity to find convincing courses concerning progress [8–10]. Fittingly, different improvements have been relied on to add to this field utilizing green cycles; for instance, the diagram of nanoparticles from plants is taken out [5, 11].

Extraction methodology is the principal headway for the store of plant metabolites from unrefined materials. To do an extraction working with exertion, some focal cutoff networks ought to be considered since these affect the chance of a concentrate [29]. The extraction of the interest packs is all through ward upon the picked part from the plant material, other than as the solvents, which need to show straightforwardness of scattering, and the delicacy to wrongly change the solutes. Since the outcome will follow from the used dissolvable, the last ought to have a low hurting penchant. Additionally, an extraction framework is picked paying exceptional brain to the length of extraction, temperature, pH of solvent, dissolvable to-test degree, and particle size of the callous materials. These parts can incite plans in the metabolite relationship of the concentrates [30]. The ideal extraction method should be the short head, less drawn-out, and finished sufficiently in any laboratory.

As proposed before, green science likes benefit over the compound, and standard method since it is unassuming, environment particularly coordinated [27], and does not need to use high beating piece, energy, temperature or ruinous made materials. Furthermore, plants have been used to produce nanoparticles since they are immediately open, found the opportunity to direct, and have an expansive drive of metabolites that can help in reduction [31]. Therefore, we expect to use innocuous to the environment and greener plans to mix gold nanoparticles. In this work, lemon peel extract was used for a mix of metal nanoparticles, and distinctive cutoff common environmental elements for saving GNPs were examined for most principal GNP creation. After that, the materials were depicted using Visual information, UV-Visible spectroscopy evaluation, TEM assessment, FT-IR examination, and SEM-EDAX appraisal. In this evaluation, lemon peel extract was used as a reductant for reducing of HAuCl_4_ solution [32]. This potential aftereffect of lemons is a rich wellspring of citrus discard and ascorbic acid [33, 34]. In like manner, the other reason for choosing lemon is a commonly available fruit.

## 2. Experiment

### 2.1. Chemicals and Materials

Chemicals and materials are chloral auric acid (HAuCl_4_ 3H_2_O, 99.9%) and the filter paper by Whatman with a pore size of 25 *µ*m and a diameter of 15 cm. The chemicals are procured from Sigma Aldrich (Poole, UK), and the lemon is collected from the local market (Taif, KSA). Distilled water is best used for this purpose.

### 2.2. Instrumentation

Instruments are a hot plate from VWR International LLC (West Chester, PA, US), transmission electron microscopy from the JOEL Ltd. (Welwyn Garden City, UK), a UV spectrophotometer from the GENESYS (Toronto, Canada), and an oven from the GALLI company (Milano, Italy). The FT-IR spectra from the PerkinElmer with the diamond ATR and energy dispersive analysis performed X-ray for the INCA 350 EDX systems from the Oxford Industry (Abingdon, UK). A sonicator from the Ultra wave Sonicator 300HD (Cardiff, UK) was used. A scanning electron microscope from Cambridge S360 from Cambridge Instrument Company (Cambridge, UK) and the Perkin Elmer 785A UV/Visible Detector from the Perkin Elmer (California, USA) were utilized.

### 2.3. Preparation of Gold Nanoparticles

Green synthesis is used with the lemon peel extract of 20 g with distilled water for 100 ml. The mixture then boiled at 80°C for 5 minutes. The solution is then filtered with the Whatman paper, and an additional 2 mM of HAuCl_4_ solution is added for a ratio of 1 : 1 at room temperature (25 ± 3°C), with stirring and kept at a dark place for 24 hours to ensure the extraction occurs. The reduction of gold ions into gold nanoparticles proceeded by a color change and the formation of the particles as observed [35, 36]. The color change is monitored in real-time to track the evolution and construction of the gold nanoparticles.

Some of the effects of the reaction, like the concentration of the lemon peel extract and the HAuCl_4_ solution, and the reactants ratio are studied to maximize the yield of the GNPs. The experiment was also performed in triplicate for reproductivity. The resulting solutions are also monitored using the UV-V spectrophotometer.

#### 2.3.1. Effect of Lemon Peel Concentration

The procedure described above is performed at different concentrations of the lemon peel extract to increase the amount of the formed GNPs, and their concentrations include 20, 40, 60, 80, and 100%.

#### 2.3.2. Effect of HAuCl_4_ Solution Concentration

The proceeding factors investigated are performed through different concentrations of HAuCl_4_ solutions of (0.5, 1.0, 1.5, 2.0 & 2.5 mM). These were investigated in order to optimize the concentration of HAuCl_4_ solution.

#### 2.3.3. Effect of Lemon Peel Extract and HAuCl_4_ Solution Ratio

Different ratios of HAuCl_4_ solution and lemon peel extract (1 : 1, 1 : 2, and 2 : 1) were investigated in order to find the maximum production of GNPs.

### 2.4. Characterization of the Formed GNPs

#### 2.4.1. Visual Observation and UV-Visible Spectra Analysis

The naked eye can observe the color of the solution under the formation of the nanoparticles. The reduction of the mixture of HAuCl_4_ solution during the exercise to extract the lemon was quickly made by the exposure to the correct formulation of the solution. The distilled water is also observed under the distilled water, and the UV-V spectrum is taken from a 1 ml sample for the distilled water. The result of the absorption is monitored spectrophotometrically. The GNPs plasmon resonance is also found in 529 nm to 500 nm in the electromagnetic spectrum [37, 38].

#### 2.4.2. TEM Analysis

The formation of gold nanoparticles was confirmed by using the electron microscope transmission. 5 *µ*l sample of the solution was put on the lacy carbon coated with 3 mm diameter copper grids. The TEM images were acquired with the Gatan UltraScan 4000 digital cameras attached to the JEOL 2010 transmission electron microscope running at 20 kV.

#### 2.4.3. SEM- EDAX Analysis

The scanning Electron Microscope of the model JSM–5800 was used to determine the shape of the developed nanoparticle. Samples were prepared by depositing drops of colloidal soliton on an aluminum grid sample holder and drying the room temperature. The elemental composition of the sample was also analyzed using the X-ray spectroscopy (EDAX) coupled to the SEM instrument.

#### 2.4.4. FT-IR Analysis

The Fourier transform infrared spectrum of the sample was recorded by an FTIR spectrophotometer. They ranged from 4000 to 500 cm^−1^ through the making of a KBr pellet with GNPs at a resolution of 4 cm^−1^.

## 3. Results and Discussion

### 3.1. Formation of GNPs

The formation of GNPs by the citrate reduction method can be summarized in three steps: reduction of Au^3+^ in solution, disproportionation of Au^+^ to gold atoms and their nucleation, growth by disproportionation on particle surface, and coagulation. Oxidation of citrate results in the formation of dicarboxy acetone, aiding nucleation [39].

The HAuCl_4_ salt disproportionates when added to water followed by ionization of the citric acid. Then, citrate will be oxidized to form dicarboxy acetone.

(1) $$\begin{array}{c} {\text{HAuCl}}_{4}+{\text{H}}_{2}\text{O}⟶{\text{AuCl}}_{3}+{\text{H}}^{+}+{\text{Cl}}^{-} \end{array}$$

(2) $$\begin{array}{c} {\text{C}}_{3}{\text{H}}_{5}\text{O}{\left({\text{CO}}_{2}\text{H}\right)}_{3}+{\text{H}}_{2}\text{O}⟶{3\text{H}}_{3}{\text{O}}^{+}+{\text{C}}_{3}{\text{H}}_{5}\text{O}{\left(\text{COO}\right)}_{3}^{-3} \end{array}$$

(3) $$\begin{array}{c} {\text{C}}_{3}{\text{H}}_{5}\text{O}{\left(\text{COO}\right)}_{3}^{-3}⟶{\text{C}}_{3}{\text{H}}_{4}\text{O}{\left(\text{COO}\right)}_{2}^{-2}+{\text{CO}}_{2}+{\text{H}}^{+}+2{\text{e}}^{-} \end{array}$$

This is followed by the reduction of auric salt to aurous salt, and then the aurous species are disproportionate to gold atoms. In the last step, the Au atoms adsorb Au^+^ complexes and form large aggregates, which leads to the formation of aggregates, by complexation with dicarboxy acetone [40, 41].

(4) $$\begin{array}{c} {\text{AuCl}}_{3}+2{\text{e}}^{-}⟶\text{AuCl}+2{\text{Cl}}^{-} \end{array}$$

(5) $$\begin{array}{c} 3\text{AuCl}⟶2{\text{Au}}^{0\;}+{\text{AuCl}}_{3}\; \end{array}$$

The overall stoichiometry of the reduction reaction can then be represented:

(6) $$\begin{array}{c} 2{\text{AuCl}}_{3}+3{\text{C}}_{6}{\text{H}}_{5}{\text{O}}_{7}^{-}⟶2{\text{Au}}^{0\;}+3{\text{C}}_{5}{\text{H}}_{4}{\text{O}}_{5}+6{\text{Cl}}^{-}+{\text{H}}^{+}+3{\text{CO}}_{2} \end{array}$$

**Scheme 1:**
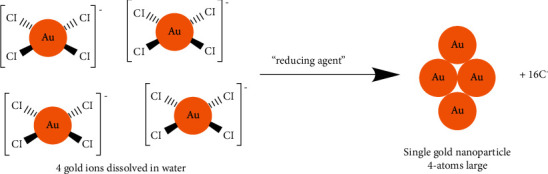
Formation mechanism of GNPs synthesized by lemon peel extract as a reducing agent.

GNPs are usually produced by the addition of a reducing agent to a solution of chloroaurate ions (AuCl_4_^−^), causing reduction of the gold ions and aggregation of the Au atoms into GNPs (Scheme 1).

### 3.2. Optimization Preparation of GNPs

#### 3.2.1. Effect of Concentration of Lemon Peel Extract

The main factor investigated was the concentration of the lemon peel extract. The different lemon peel extract percentages (20, 40, 60, 80, and 100%) was used for the best creation of GNPs and to ensure that reduced gold salt was present in the solution.

**Figure 1:**
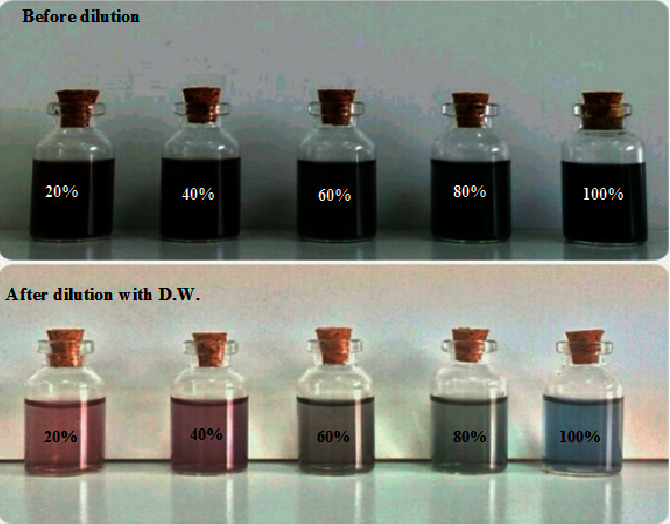
The pictures show the color changes after the process of reduction of HAuCl_4_ solution to GNPs using different concentrations of lemon peel extract before and after dilution with distilled water.

**Figure 2:**
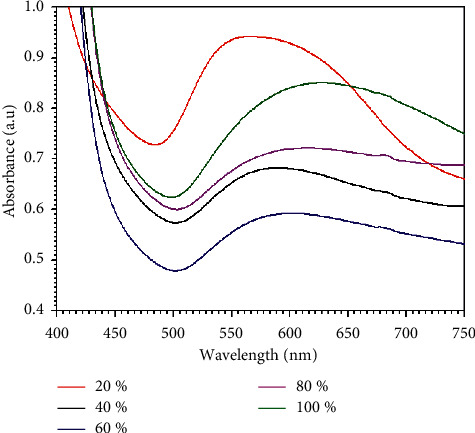
UV-Vis absorption spectra of GNPs solution using different concentrations of lemon peel extract.

The color of the form solution was observed using a self-administering eye to check the formation of GNPs. It was tracked down that the shadow of the fluid outline of HAuCl_4_ changed from yellow to the toned arrangement in the wake of adding the lemon peel extract, as can be found in Figure 1. Additionally, the advancement of reducing of HAuCl_4_ technique response with metal particles inside seeing lemon peel extract concentrate can be reasonably evaluated by UV-Vis spectra of gold nanoparticles in fluid blueprint utilizing a UV-Visible spectrophotometer. This is considering how gold nanoparticles can show optical properties identified with Localized Surface Plasmon Resonance (LSPR), which depends on the morphology of the nanoparticles [36]. The UV-Vis spectrograph of the colloidal arrangement of GNPs was recorded with purified water as a blank. The impact of the lemon peel extract turning around creating gold nanoparticles was reviewed spectrophotometrically after 48 h. As can be found in Figure 2, the highest absorption peak was seen while utilizing 20% of lemon peel extract, showing that the most unbelievable concentration of lemon peel extract concentrates of 20% is extraordinarily cautious for the formation of GNPs by lemon peel extract.

While utilizing 20% concentration of lemon peel extract, the nanoparticles formation and size reduction were begun rapidly because of the more availability of functional groups in the lemon peel extract.  Table 1 sums up the impact of various concentration of lemon peel extract in the nanoparticle's preparation. After 10 min, the color of the mixture changes from yellow to ruby red, grey or blue color for the formed GNPs utilizing different concentration of lemon peel extract at room temperature. The higher speed of reduction happened at lower concentration of the reducing reagent (20% of lemon peel extract). The broadening peak was gotten at 60% of lemon peel extract showing the sized nanoparticles. The narrowest peak was obtained at 20%, which shows the laid-out nanoparticles are small in size and the higher speed of decreasing of gold particles happened in the 20%. At last, it was considered that lower concentration was optimum for fabrication of GNPs.

**Figure 3:**
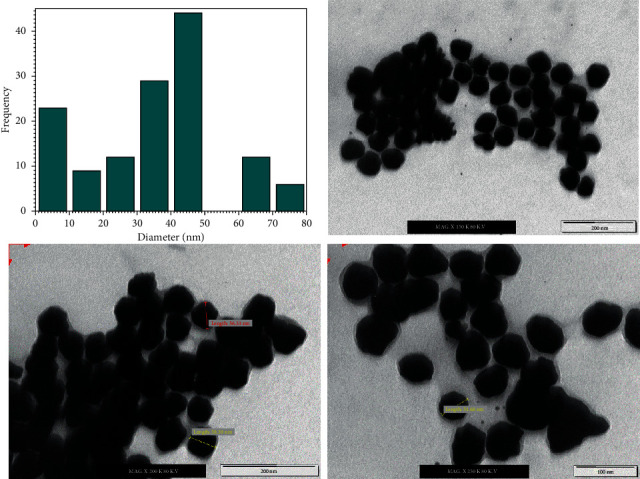
The size distribution histogram and TEM images of GNPs using optimum concentrations of lemon peel extract (20%) using different magnifications.

The size distribution of nanoparticles, all things considered, is a fundamental issue as nanoparticles show specific physical and substance properties relying upon their shape and size. TEM is in this strategy for the most adapted techniques to study the size and shape of the nanoparticles and give their distribution [42]. In this evaluation, the prepared GNPs were characterized utilizing TEM analysis.  Figure 3 shows the size distribution histogram and TEM micrographs showing the formation of nanoparticles. The TEM micrographs showed that the amount of the prepared GNPs utilizing the ideal concentration of lemon peel extract at various magnification was spherical in shape, and they were dispersed in a watery medium. It was tracked down that the size of the prepared GNPs relies upon the concentration of the lemon peel extract. It was found that the size of GNPs was 40.5 nm.

#### 3.2.2. Effect of Concentration of HAuCl_4_ Solution

**Figure 4:**
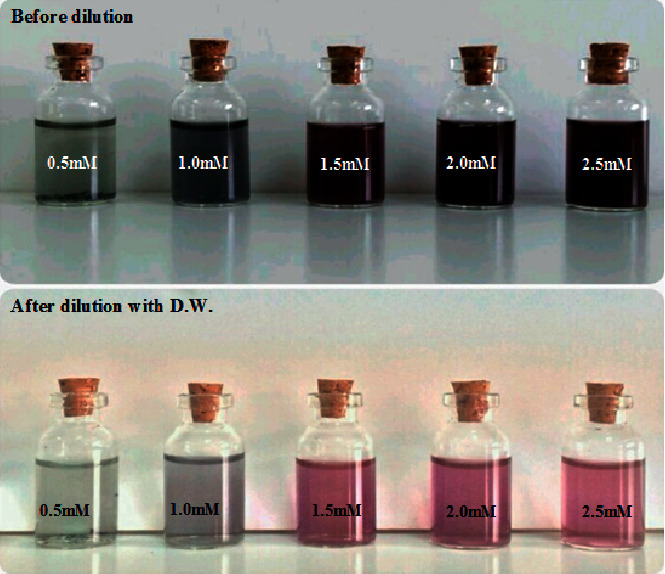
The pictures show the color changes after the process of reduction of HAuCl_4_ solution to GNPs using different concentrations of HAuCl_4_ solution (0.5, 1.0, 1.5, 2.0, and 2.5 mM) before and after dilution with distilled water.

**Figure 5:**
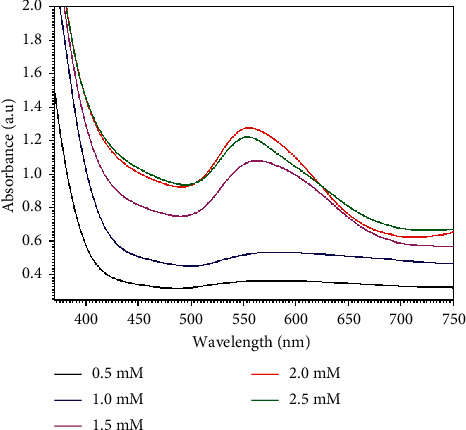
UV-Vis absorption spectra of the formed GNPs using different concentrations of HAuCl_4_ solution.

After discovering of the best concentration of lemon peel extract that was 20%, different concentration of HAuCl_4_ solution (0.5, 1.0, 1.5, 2.0, and 2.5 mM) were utilized to maximize the yield of GNPs. The color of the mixture was seen by naked eye to check the formation of GNPs. It was found that the color of the aqueous solution of HAuCl_4_ changed from yellow to colored solution after adding the lemon peel extract, as can be seen in Figure 4, which presents photographs showing the changing color of the solution using different concentration of the HAuCl_4_ solution for preparation of GNPs. The reason for the color change is the reduction of the gold ions. As can be seen in Figure 5, the UV-Visible absorption spectra showed that by increasing the concentration of HAuCl_4_ concentration, the absorbance of GNPs was improved, and the best absorbance of GNPs was extended when the concentration of HAuCl_4_ solution was 2 mM. Increasing intensity of the peak indicated increasing the amount of the formed nanoparticles and higher concentration of gold solution suggests the preparation of larger nanoparticles [43].

**Figure 6:**
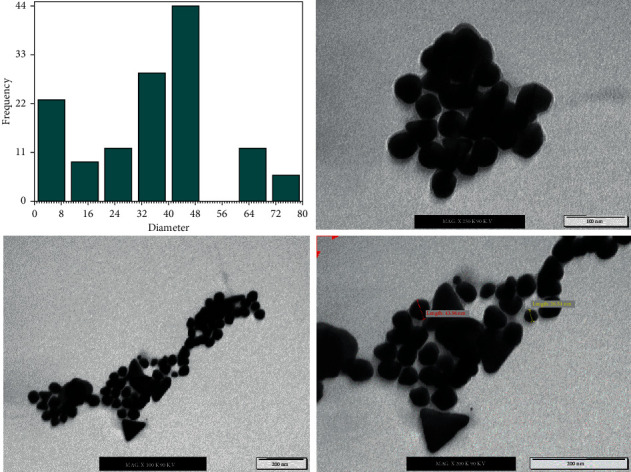
The size distribution histogram and TEM images of GNPs using optimum concentrations of HAuCl_4_ solution (2 mM) using different magnifications.

Table 2 summaries the color, absorbance, and wavelength of the prepared GNPs using different concentration of HAuCl_4_ solution. After addition of the reducing reagent to the HAuCl_4_ solution, the color changes from yellow to grey or ruby red color for the arranged GNPs in a brief timeframe at room temperature. The absorption was extended while increasing the concentration of the gold ions from 0.5 mM to 2 mM, and by using concentration of 2.5 mM of HAuCl_4_ solution, the absorbance of the prepared GNPs was reduced. Fittingly, the optimization study showed a significant effect of the HAuCl_4_ solution on the synthesis of GNPs. This investigation sees that the best HAuCl_4_ solution was 2 mM for nanoparticles preparation. The prepared GNPs using ideal concentration of the HAuCl_4_ solution (2 mM) were depicted using TEM analysis.  Figure 6 shows the size distribution histogram and TEM electron microscopy micrographs showing the prepared gold nanoparticles. The TEM micrographs showed that the extent of the made GNPs using the optimum concentration of HAuCl_4_ solution at different magnifications was spherical and dispersed in a watery medium. It was found that the size of the prepared GNPs depends on the concentration of the HAuCl_4_ solution. The size of the prepared GNPs was 38.42 nm when the concentration of the HAuCl_4_ solution was 2 mM.

#### 3.2.3. Effect of Ratio of HAuCl_4_ Solution and Reducing Reagent

**Figure 7:**
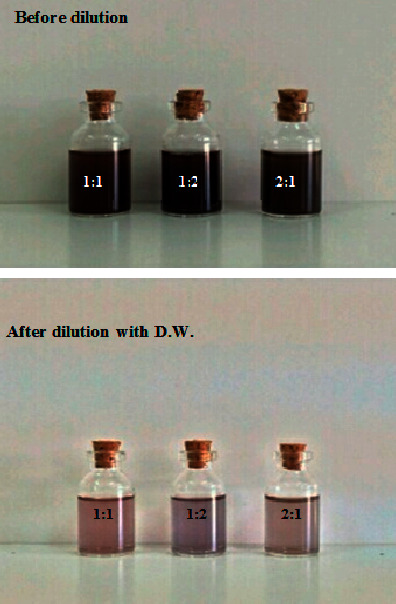
The pictures show the color changes after the process of reduction of HAuCl_4_ to GNPs using different ratios of HAuCl_4_ solution to lemon peel extract (1 : 1, 1 : 2, and 2 : 1) before and after dilution with distilled water.

**Figure 8:**
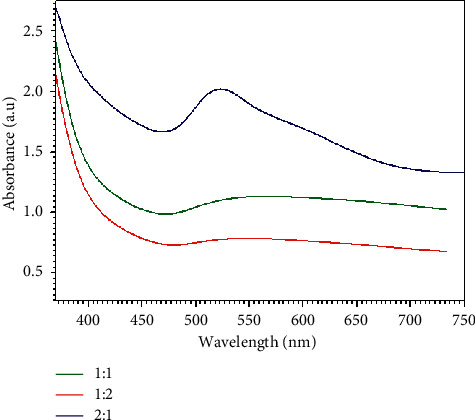
UV-Vis absorption spectra of GNPs using different ratios of HAuCl_4_ solution (2 mM) to lemon peel extract (20%).

The concentration of lemon peel extract (20%) and HAuCl_4_ solution (2 mM) in the ranges of 1 : 1, 1 : 2, and 2 : 1 was utilized to find the optimum composition of the preparation of GNPs since reactants ratio is an important factor affecting the formation of GNPs and even nanoparticles sorting out [44]. GNPs were prepared using different ratio of HAuCl_4_ solution to lemon peel extract. On mixing the reducing reagent with the aqueous solution of HAuCl_4_, the color of the mixture was changed from yellow to colored solution, as can be seen in Figure 7. The color change indicated the reduction of the chloroauric acid ions using lemon peel extract, forming GNPs. From Figure 8, it was found that the best ratio to get a high absorbance of GNPs was when the ratio of HAuCl_4_ solution and lemon peel extract in the reaction mixture was 2 : 1 based on the number of trials and the best yield.

Table 3 presents the effect of ratio of HAuCl_4_ solution and lemon peel extract. The color changes from yellow to ruby red for 1 : 1, 1 : 2, and 2 : 1. The UV spectra show the SPR band in the space of 523 nm, and the absorbance was 2.023 a.u. (Figure 8). Contemplating everything, the best condition for preparation of GNPs when using 20% of lemon peel extract, 2 mM of HAuCl_4_ solution, and ratio of HAuCl_4_ solution to lemon peel extract was 2 : 1.

**Figure 9:**
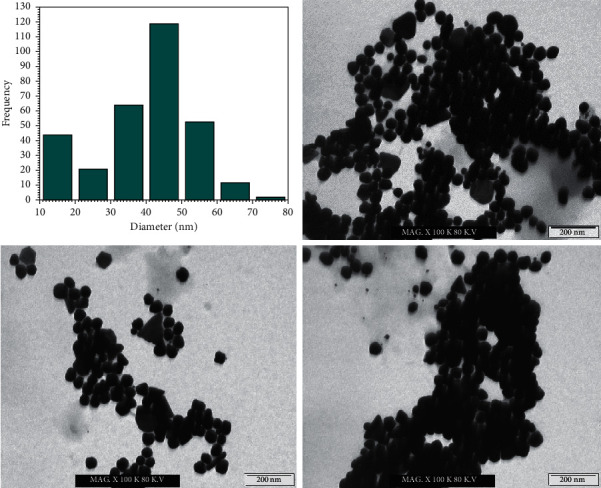
The size distribution histogram and TEM images of GNPs using optimum ratio of HAuCl_4_ solution (2 mM) to lemon peel extract (20%) that was 2 : 1.

The prepared GNPs using optimum ratio (2 : 1) of HAuCl_4_ solution (2 mM) to lemon peel extract (20%) were depicted using TEM analysis, since it can give information about the morphology and size of the prepared nanoparticles.  Figure 9 shows the size distribution histogram and TEM micrographs showing the formation of nanoparticles. The TEM micrographs showed that the prepared GNPs were spherical. The size of GNPs was measured by measuring the diameter of whole particles on TEM images. The average diameter of prepared GNPs was in the range of 25.36 nm with very few particles of higher and lower size distribution. The TEM micrographs show that most of the gold nanospheres are round or spherical in shape with a degree of faceting due to their crystalline nature.

### 3.3. Characterization of the Prepared GNPs

At whatever point a nanoparticle association cycle is performed, portraying the new material to know the morphology, size, and achievement of the synthesis process. A different technique was utilized to characterize the prepared GNPs, which were SEM, EDAX, and IR. SEM framework can give the shape, size, and surface morphology of nanoparticles [45].

**Figure 10:**
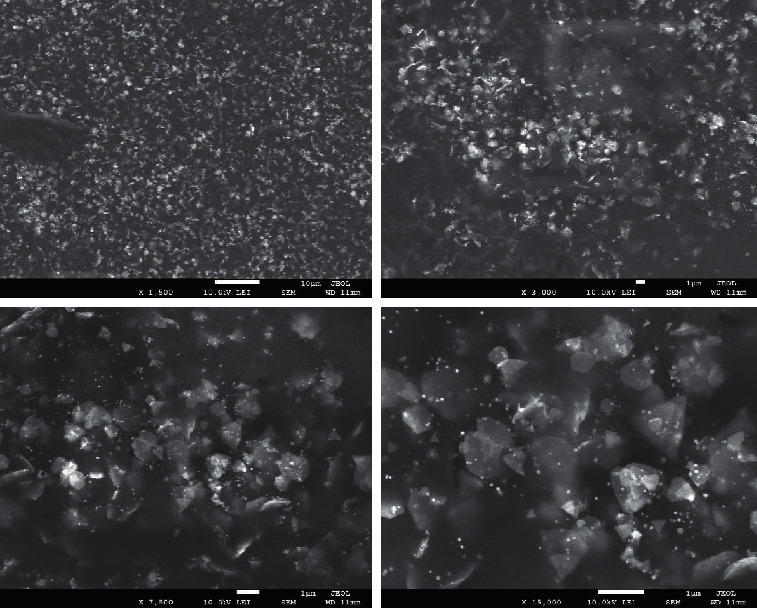
SEM images of the synthesized GNPs using different magnifications.

From SEM experiences as displayed in Figure 10, the surface morphology of the coordinated GNPs wires everything consisting of spherical particles. Minority amount of nonround particles is also viewed, all around nanoprisms and nanotriangles. It is well known that particles have different sizes, from 10 nm to 100 nm, so they are not monodispersed. The surface is unnecessarily covered, likely by citrate as stabilizing agent. Aggregation of particles can be seen and size of the mass is around 1 micron.

**Figure 11:**
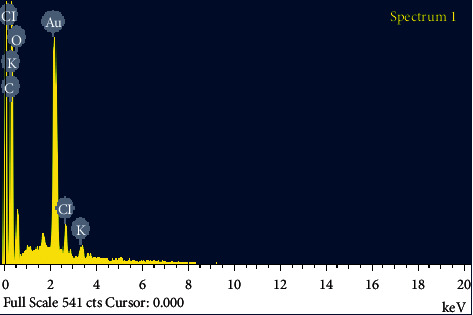
EDAX image of the synthesized GNPs.

The green-fabricated GNPs were assessed further utilizing EDAX spectrometry, which upheld gold without any contaminants. The optical adsorption peak was typically seen at 2.30 keV (Figure 11), which is the ordinary adsorption of gold nanocrystallites because of surface plasmon resonance. The EDAX profile for the prepared GNPs showed solid gold molecule signals at around 2.30 keV. EDAX for the GNPs offered substantial clues for gold particles and weaker signals for carbon, oxygen, and chloride, prevenient from the lemon peel's biomolecules. The strong signals signs are gotten at around 2.30 keV [46, 47].  Table 4 presents the atomic percentages of the nanoparticle, which were gold, carbon, oxygen, potassium, and chloride. It was tracked down that the Au atomic was on a fundamental level of 6.17%.

**Figure 12:**
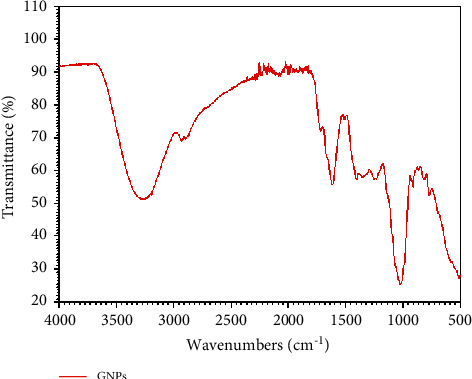
FT-IR spectra of the synthesized GNPs.

FT-IR spectrum identified the functional groups which are involved in the gold nanoparticles synthesis.  Figure 12 shows FT-IR spectra of GNPs utilizing lemon peel extract as a reducing reagent while Table 5 presents the experimental wavenumber, their assignments, and the wavenumber limits for the integration. It can be seen peaks at 3266 cm^−1^, 2935 cm^−1^, 1614 cm^−1^, 1406 cm^−1^, 1258 cm^−1^, and 1023 cm^−1^. From the outcomes, it could be inferred that the C. limon pectin shows sharp and strong peaks at 3266.25 cm^−1^ as O-H stretch, C-H stretch in the frequency 2935 cm^−1^ displayed as carbohydrate ring [48, 49]. The absorption peaks 1023 cm^−1^ were from the ether (C-O-C) [50], C-H scissoring and bowing conduct is displayed by a top between 1406 cm^−1^ [51], peak for group C-O stretching vibrations is coordinated in 1258 cm^−1^, and at last peak credited to group C=C stretching was located at 1614 cm^−1^ [52].

## 4. Conclusion

Green synthesis is an alternative way for preparation of gold nanoparticles since it is environmentally friendly, safe, and available. Several parameters are used for the optimum conditions to prepare gold nanoparticles that were concentration of lemon peel extract, concentration of HAuCl_4_ solution, and ratio of HAuCl_4_ solution to lemon peel extract. A surface Plasmon resonance typically has a peak from 535 to 579 nm confirming the gold nanoparticles synthesis. Additionally, different characterization techniques were used to study the formed metal nanoparticles such as SEM, EDAX analysis, and FT-IR spectroscopy. The spherical sized nanoparticles were confirmed; moreover, size distribution and morphology of the GNPs were characterized in detail. The results of the investigation showed that the optimum condition for preparation of GNPs using lemon peel extract was 20% of lemon peel extract, and 2 mM of HAuCl_4_ solution and ratio of HAuCl_4_ solution to lemon peel extract was 2 : 1. The method was simple to perform and yielded stable GNPs. The synthesized GNPs using lemon extract can be exploited towards the development of potential antibacterial agents.

## Figures and Tables

**Table 1:** Synthesis of GNPs using different concentrations of lemon peel extract.

Concentration of HAuCl_4_ solution (mM)	Concentration of lemon peel extract (%)	Ratio	Color of solution	Absorbance (nm)	SPR band (nm)
2.0	20	1 : 1	Ruby red	0.941	566
2.0	40	1 : 1	Ruby red	—	—
2.0	60	1 : 1	Grey	—	—
2.0	80	1 : 1	Grey	—	—
2.0	100	1 : 1	Blue	—	—

**Table 2:** Synthesis of GNPs using different concentrations of HAuCl_4_ solution.

Concentration of HAuCl_4_ solution (mM)	Concentration of lemon peel extract (%)	Ratio	Color of solution	Absorbance (a.u)	SPR band (nm)
0.5	20	1 : 1	Grey	—	—
1.0	20	1 : 1	Grey	—	—
1.5	20	1 : 1	Ruby red	1.082	548
2.0	20	1 : 1	Ruby red	1.279	542
2.5	20	1 : 1	Ruby red	1.223	539

**Table 3:** Synthesis of GNPs using different ratios of HAuCl_4_ solution to lemon peel extract.

Concentration of HAuCl_4_ solution (mM)	Concentration of lemon peel extract (%)	Ratio	Color of solution	Absorbance (a.u)	SPR band (nm)
2.0	20	1 : 1	Ruby red	—	—
2.0	20	1 : 2	Ruby red	—	—
2.0	20	2 : 1	Ruby red	2.023	523

**Table 4:** EDAX elemental microanalysis of the synthesized GNPs.

Element	Weight%	Atomic%
C K	39.44	80.83
O K	6.32	9.73
Cl K	3.22	2.24
K K	1.64	1.03
Au M	49.38	6.17
Totals	100.00	100.00

**Table 5:** Assignment of bands observed in the FT-IR spectrum of the synthesized GNPs.

Experimental wavenumber (cm^−1^)	Assignment	Range (cm^−1^)
2935	C-H stretch	2950-2800
1406	C-H in-plane bend C=C	1430-1290
1614	Stretch (conjugated)	1640-1610
1023	C-O stretch	1260-1000
3266	O-H stretch	3550 - 3200

## Data Availability

The data used in this paper are available upon reasonable request from the corresponding author.

## Additional Points

No animals/humans were used for studies that are the basis of this research.
